# The Allen’s test: revisiting the importance of bidirectional testing to determine candidacy and design of radial forearm free flap harvest in the era of trans radial endovascular access procedures

**DOI:** 10.1186/s40463-015-0096-0

**Published:** 2015-11-04

**Authors:** Andrew Foreman, John R. de Almeida, Ralph Gilbert, David P. Goldstein

**Affiliations:** Department of Otolaryngology Head and Neck Surgery, University Health Network, Princess Margaret Cancer Centre, University of Toronto, Toronto, ON Canada

**Keywords:** Allen’s test, Radial artery, Reconstruction, Head and neck cancer, Endovascular

## Abstract

**Background:**

The radial forearm free flap is a workhorse free flap. The radial artery, which supplies it, is increasingly being used for endovascular access. A complication of this is radial artery occlusion. Although often asymptomatic it can compromise future free tissue transfer.

**Case Presentation:**

Two patients who underwent RFFF harvest for head and neck reconstruction are presented; both of who likely had distal radial artery occlusion.

The first patient had failure of flap perfusion, presumed secondary to radial artery occlusion from prior endovascular access at the distal radial artery. In the second case, we used the Allen’s test in reverse to identify the same scenario and successfully redesigned the harvest.

**Conclusion:**

The Allen’s test is a simple bedside test that should be performed bidirectionally to exclude radial artery occlusion, which may compromise flap harvest. Radial artery occlusion will become increasingly common as the radial artery is used more frequently for endovascular access procedures.

## Background

**Fig. 1:**
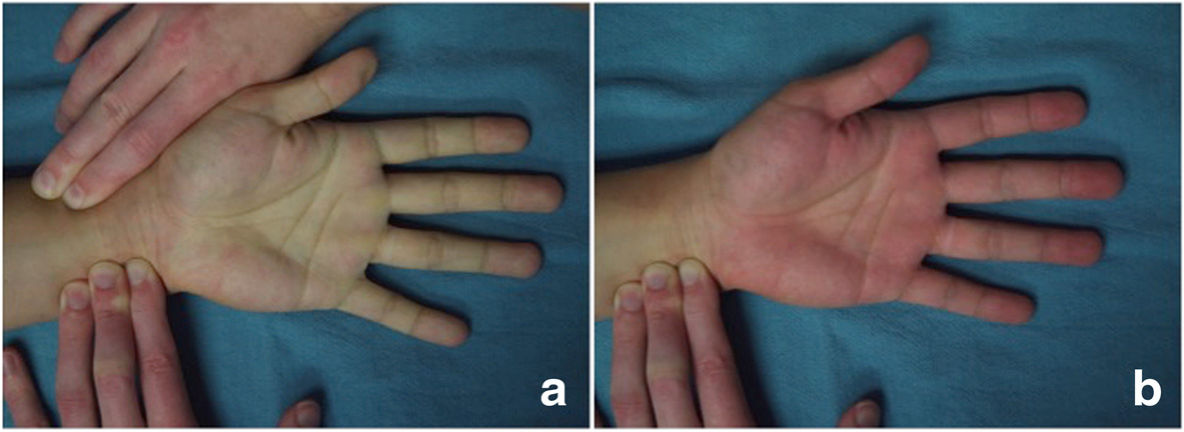
The ‘reverse’ Allen’s test. **a**. The pallor associated with occlusion of both radial and ulnar artery inflow to the hand. **b**. Rapid return of redness to the hand following release of radial artery pressure confirming flow through the radial artery

First described in 1929 by Edgar Allen, the Allen’s test has become the most common method for assessing palmar arch patency [1]. Allen originally described his test for diagnosis of thromboangiitis obliterans of the ulnar artery however more recently it has been used to assess the adequacy of the ulnar collateral blood flow through the palmar arches prior to radial artery sacrifice during cardiac surgery and reconstructive surgery [2]. As a pre-operative test it provides a degree of safety in preventing hand ischemia prior to undertaking these procedures. Its reported sensitivity and specificity are 54.5 and 91.7 % respectively, which is acceptable given the simple and non-invasive nature of the test [3]. Given the dual arterial inflow to the hand, the Allen’s test can be performed in both directions to assess either ulnar or radial artery patency individually. Whilst most commonly used to assess collateral flow through the ulnar artery, we believe this bedside assessment also has utility when performed in reverse to assess the flow through the radial artery (Fig. 1).

Cannulation of the radial artery for invasive monitoring and interventional procedures is becoming increasingly common. It places a significant number of patients at risk for asymptomatic radial artery occlusion, which may compromise subsequent radial forearm free flap (RFFF) harvest. We present a case of a compromised RFFF that was believed to be due to arterial injury from a prior radial arterial line and a second case where bidirectional Allen’s testing was able to pre-operatively identify a patient with radial artery occlusion that led to a change in RFFF design. These cases highlight the importance of performing the Allen’s test in both directions prior to RFFF harvest to not only prevent hand ischemia but also to ensure adequacy of arterial inflow to the flap harvest site.

## Case Presentation

### Case Presentation: Illustrative case 1

A 66-year-old female presented to our head and neck clinic with a 2.5 cm left floor of mouth, biopsy-proven squamous cell carcinoma. The treatment recommendation was for primary surgery consisting of floor of mouth excision, bilateral selective neck dissections and radial forearm free flap (RFFF) reconstruction.

Her past history was significant for a colonic perforation approximately 2 years prior to her presentation to our clinic. At that time she required a laparotomy and colostomy followed by an extended intensive care unit stay. She was right-hand dominant and denied any trauma or surgery to the left forearm or hand. She was a 50-pack year smoker and a heavy alcohol drinker. Pre-operatively she was assessed for RFFF harvest with a standard Allen’s test and pulse oximetry plythesmography, which were both normal. She had a palpable radial pulse and no visible evidence of trauma at the donor harvest site.

She was taken to the operating room and underwent resection of the primary site. A RFFF measuring 5 × 4 centimeters was designed overlying the radial artery with the distal aspect of the skin paddle placed approximately 3 cm proximal to the left flexor wrist crease. The flap was raised in standard fashion. During the flap elevation, significant fibrosis was encountered at the distal aspect of the pedicle as well as along the flexor retinaculum underlying the pedicle.

After the tourniquet was released, the skin paddle of the flap was not perfused. Assessment of the radial artery with pencil Doppler confirmed a pulse in the proximal artery but this was lost at the proximal end of the skin paddle. No pulse was obtainable within the borders of the flap. The distal artery clip was removed and there was no flow-through observed. A Fogarty catheter was then inserted from the distal end of the radial artery, however there was significant resistance towards the proximal end of the flap suggesting stenosis of the vessel. The catheter was passed proximal to the stenosis and on inflation and withdrawal of the catheter flow through the flap was able to be re-established however this was not sustained. A decision was made at this time to abort the RFFF. A left anterolateral thigh flap was raised without complication and used as reconstruction.

**Fig. 2:**
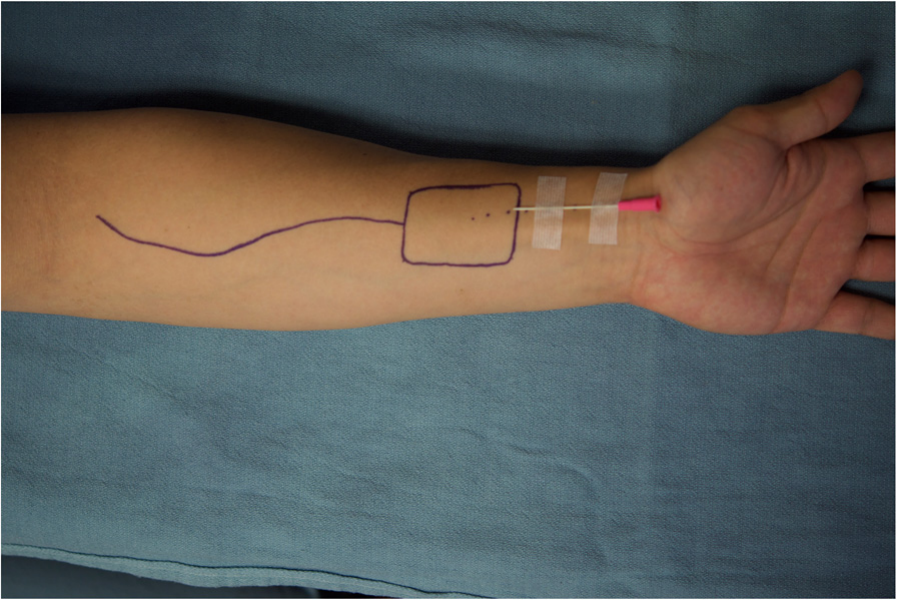
Redesigning a more proximal radial forearm free flap avoids the area of likely vessel trauma from an intra-arterial cannula. In this image, a standard intra-arterial cannula is positioned overlying the distal radial artery. The exact length of stenosis can be confirmed with Doppler ultrasonography

We hypothesized that during the prior surgery and ICU admission for management of her colonic perforation she had a left radial artery intra-arterial catheter placed for hemodynamic monitoring. This likely resulted in occlusion of the left radial artery, which was not appreciated on pre-operative assessment. Interestingly the location and length of radial artery occlusion in our case corresponds to the length of a standard intra-arterial catheter from its usual insertion site at the flexor wrist crease, as illustrated in Fig. 2.

### Illustrative case 2

Following the first case we encountered a 69 year-old patient with an oral tongue cancer that required free-tissue reconstruction. He had a history of multiple medical comorbidities including diabetes, chronic renal failure and peripheral vascular disease, in addition to a history of prior surgeries. He had an arteriovenous fistula in his right arm and a left leg below-the-knee amputation. The Allen’s test on the left hand was normal. We then assessed the patency of the radial artery by performing the Allen’s test in reverse. Just as the radial artery occlusion is maintained with digital pressure in a standard Allen’s test to assess the ulnar artery supply to the arches, palmar inflow through the radial artery is assessed in the ‘reverse’ Allen’s test by maintaining ulnar artery compression. With normal radial artery patency the tester can expect rapid return of color to the hands and fingers upon release of the radial artery as illustrated in Fig. 1. In this case there was no evidence of hand re-perfusion upon release of the radial artery. While maintaining ulnar compression the radial artery was assessed with the hand-held Doppler, which demonstrated that there was no Doppler signal of the radial artery at the flexor crease, however a Doppler signal was present more proximal on the artery, approximately 5 to 6 cm from the flexor crease. We therefore designed our flap in a more proximal location, an example of which is demonstrated in Fig. 2.

Flap harvest in this way was successful with a well-perfused flap transplanted to the hemi-glossectomy defect. The long pedicle length of the RFFF is advantageous in this situation, whereby shortening the pedicle length by moving the donor harvest site more proximal on the arm still permitted arterial and venous anastomoses to be performed in the neck without the need for vein grafts or deferring to an alternate donor site.

The RFFF has established itself as a workhorse reconstructive option for a multitude of head and neck defects providing thin, pliable tissue supplied by a long, large caliber vascular pedicle. Furthermore its attractiveness is enhanced by it being a reliable donor site with flap failure rates reported to be less than 3 % and an anatomical location that permits two-team harvest [4–6]. There are, however, potential morbidities associated with this flap harvest [7]. These may include a cosmetically displeasing donor site closure, skin graft loss with subsequent flexor tendon exposure along with alterations in range of movement, strength and sensation in the donor hand and forearm [8]. Despite their rarity, the most feared complications of RFFF harvest are the ischemic hand complications [9].

The vascular supply of the hand is derived from the superficial and deep palmar arches, which receive their arterial inflow from the radial and ulnar arteries. The ulnar artery is usually the dominant contributor to the superficial arch, anastomosing with the superficial branch of the radial artery over the thenar eminence [10]. Palmar digital arteries run distally from this arch to supply the fingers. A complete superficial arch is present in 84–90 % of patients, with considerable variation occurring. In contrast, the radial artery predominantly supplies the deep arch. It almost invariably forms a complete arch through anastomosis with the deep branch of the ulnar artery. The palmar metacarpal arteries arise from the deep arch and anastomose with the palmar digital arteries from the superficial arch. Hence the hand is supplied by an anastomosing network of arteries arising from both superficial and deep palmar arches, which in turn are supplied from a combination of the radial and ulnar arteries. These extensive anastomotic connections usually prevent the hand from ischemic damage in the face of injury to a single component of the network.

Following harvest of the radial artery during the RFFF harvest, the hand is solely perfused by the ulnar supply to the arches and the distal anastomotic connections ensure the hand and fingers remain perfused. However, inadequate flow through the ulnar artery may result in either acute or chronic hand ischemia [9]. The adequacy of flow through the palmar arches is routinely tested pre-operatively with the Allen’s test to pre-emptively identify patients at risk of hand ischemia after radial artery sacrifice. Other tests such as Doppler ultrasonography and pulse oximetry with plythesmography have also been employed to improve accuracy of pre-operative clinical decision-making [11]. This dedicated assessment of the adequacy of the arch system reflects the seriousness of the morbidity should inadequate arch circulation be overlooked prior to RFFF harvest.

In contrast to the attention given to the potentially devastating donor site morbidity related to inadequate ulnar inflow, little has been published on the impact of radial artery occlusive disease in the setting of attempted RFFF harvest. The very nature of the arterial arcades within the hand may mask pre-existing radial artery damage as ulnar collateral circulation through the palmar arches can produce a palpable pulse on the radial side of the wrist crease in spite of radial artery occlusion more proximally. This may predispose to intra-operative flap failure. Performing the Allen’s test in the reverse direction can ensure the adequacy of flow through the radial artery and prevent this potential complication.

Recently there has been increasing use of invasive monitoring during anesthesia and the intensive care setting as well as a dramatic expansion of therapeutic endovascular procedures. The radial artery has become an attractive option for arterial catheterization due to its superficial location and fewer access site complications. Whilst the complications of trans radial catheterization are reported to be lower than other sites, radial artery occlusion is the most frequently encountered [12]. The reported incidence of radial artery occlusion is 2 to 18 % [13], however it is recognized this may be an under-representation because this condition is usually asymptomatic and only detected with ultrasound or plethysmographic assessment of the radial artery or when the radial artery is re-accessed for another endovascular procedure.

As with ulnar artery disease, radial artery occlusion may be a silent disease given the arcade-like nature of the palmar vascular anatomy. Quite rightly, the vascular literature concludes that this condition is associated with essentially no major clinical sequelae, however it does limit the future utility of the radial artery as an arterial access site [14]. It is important to recognize that this also extends to its use in a free tissue transfer. It is interesting to note that the length of an intra-arterial catheter from the usual insertion site at the wrist corresponds to the area most commonly used to harvest a RFFF (Fig. 2).

As the use of the radial artery for diagnostic and therapeutic procedures continues to expand it is behest on the reconstructive surgeon to consider this potentially silent disease in planning head and neck reconstruction. To this end, we reiterate the importance of performing a detailed assessment of both ulnar and radial artery blood flow prior to RFFF harvest by performing the Allen’s test in both directions, as already described. When the ‘reverse’ Allen’s test demonstrates a complete lack of re-perfusion of the hand and digits then a lack of flow through the radial artery should be assumed. This is in contradistinction to the situation where there is reperfusion to the thumb and index finger, which is due to an incomplete arch with lack of communication with the ulnar system. In the latter situation a hand-held Doppler can be used to trace the flow in the radial artery in order to determine candidacy for a RFFF and when flow is present proximally it can aid in designing the flap more proximally over an area of skin that will be perfused by the radial artery. Alternatively, another donor site may be chosen, particularly if the injury to the radial artery extends proximally.

## Conclusion

In summary, the Allen’s test is a time-honored, simple and non-invasive test to assess arterial flow through the palmar arches of the hand. It is important to remember that this is a bidirectional test and it should be performed in both directions prior to RFFF harvest. This can prevent both ischemic hand complications and ensure that radial artery inflow to the distal forearm is sufficient to ensure a RFFF will perfuse after harvest. We highlight these technical details through two illustrative cases because we believe it is increasingly relevant in the current era where the radial artery is frequently being used as an access site for invasive monitoring and therapeutic endovascular procedures. This expansion of practice places an increasing number of patients at risk of radial artery occlusion a disease that is most often silent due to the extensive anastomotic connections of the palm. Furthermore patients may not recall they have had arterial cannulae placed during their prior hospital stays rendering history alone unreliable.

## Consent

Written informed consent was obtained from the patient for publication of this case report. A copy of the written consent is available for review by the Editor-in-Chief of this journal.
